# Development and Validation in Porcine and Human Models of a Bioimpedance Spectroscopy System for the Objective Assessment of Kidney Graft Viability

**DOI:** 10.3390/s25092871

**Published:** 2025-05-02

**Authors:** David Naranjo-Hernández, Javier Reina-Tosina, Laura M. Roa, Gerardo Barbarov-Rostán, Jorge Calvillo-Arbizu, Alejandro Talaminos-Barroso, Miguel Ángel Pérez-Valdivia, Rafael A. Medina-López

**Affiliations:** 1 Biomedical Engineering Group, Department of Signal Theory and Communications, University of Seville, 41092 Seville, Spaingbarbarov@us.es (G.B.-R.); jcalvillo@us.es (J.C.-A.);; 2 Clinical Management Unit of Urology and Nephrology, Virgen del Rocío University Hospital, 41013 Seville, Spain

**Keywords:** bioimpedance spectroscopy, Cole model, kidney transplant, ischemia monitoring

## Abstract

This work presents an innovative bioimpedance spectroscopy device, developed as a support tool for decision-making during the evaluation of kidney viability for renal transplantation. Given the increasing demand for organs and the need to optimize donation criteria, the precise and objective assessment of renal graft functionality has become crucial. The device, based on a modular design and adapted to the surgical environment, uses a novel Cole model with a frequency-dependent membrane capacitance, which improves measurement accuracy and repeatability compared to conventional models. Adapting the device for operating room usege involved overcoming significant challenges, such as the need for sterilization and a visual, tactile and acoustic user interface that facilitates device usability. Optimizing the sensing stage has minimized the influence of measurement artifacts, which is crucial for obtaining accurate and representative measurements of renal tissue bioelectrical properties. In addition, a rigorous electrode sterilization protocol was designed, ensuring asepsis during the procedure. The results of tests on porcine renal models demonstrated the device’s ability to monitor pathophysiological changes associated with renal ischemia, with a notable improvement against measurement repeatability.

## 1. Introduction

In the current health landscape in Spain, over 5000 people await the opportunity for a kidney transplant [1]. In the United States, this number increases to more than 70,000 people [2]. Simultaneously, the incidence of patients requiring renal replacement therapy continues its upward trajectory [3,4]. The demand for kidney transplants far exceeds the availability of donor organs. To combat this global shortage, a wider range of deceased donor kidneys are being used [5]. Faced with this reality, recent decades have witnessed an expansion in the donation criteria, including the consideration of elderly donors. However, age emerges as a critical influencing factor in renal graft functionality, graft survival, and recipient patient survival [6,7,8]. The use of elderly donors, while necessary, introduces an additional level of complexity in the evaluation of graft viability. Therefore, the decision to accept a donor kidney must strike a balance between maximizing the use of scarce organs and minimizing the risk to the recipient [5].

The precise evaluation of renal graft viability is a determining factor in transplant success. In an effort to optimize this process, various protocols have been implemented, with pre-implantation renal biopsy being a widely used diagnostic tool. This procedure, which provides a detailed anatomopathological report, seeks to ensure organ functionality before implantation.

However, the use of this protocol has raised great controversy in recent years, mainly due to the increase in the percentage of discarded organs [9], and there is a debate about whether all these discards are truly justified [5].

Worldwide, the kidney discard rates range from 12% to 20% [10]. The United States surpasses the world average for the highest proportion of discarded kidneys from deceased donors, the rate reaching 25% in 2022 [10]. In the United States, more than half of deceased donor kidneys are biopsied, with thousands of deceased donor kidneys discarded annually due to unacceptable pre-implantation renal biopsy readings [11,12]. Additionally, biopsied kidneys are three times more likely to be discarded than kidneys that are not biopsied [5]. Although discarding a fraction of organs obtained from deceased donors is justified, some of these kidneys could be suitable for transplantation, and a large percentage (62%) could have been transplanted if a scoring system other than the American one had been used [10].

In this sense, some works have questioned biopsy-based procedures, arguing that they are poorly reproducible and are not significantly associated with transplant results [13]. In another international multicenter study, the authors concluded that the histological evaluation of the pre-implantation kidney donor did not add value to the organ allocation process, but rather resulted in discarding potentially usable organs [12].

A recent study indicated that, of 398 kidneys discarded due to unacceptable biopsy results, 27% of the discarded kidneys could have demonstrated survival similar to that of other transplanted kidneys [14]. Other authors considered that renal biopsies do not consistently predict the possibility of early graft failure [10,11,13].

Furthermore, pre-implantation renal biopsy is expensive and time-consuming [15], which is even more valuable because the time lost waiting for the result prolongs the cold ischemia time, which is associated with a worse prognosis, an increased risk of organ deterioration [16], and ultimately organ non-implantation [17].

This situation can be attributed, to a large extent, to the inherent subjectivity of the biopsy procedure [11,18,19,20,21]. Inter-observer variability in the interpretation of biopsy results, the lack of uniformity in renal tissue processing techniques for histological analysis, and the trend to overestimate glomerulosclerosis are factors that contribute to evaluation inconsistency [22]. Furthermore, the scoring system, which heavily relies on specialist criteria, adds an additional subjective component due to variability in pathologists’ experience [18]. These differences make it difficult to compare studies and limit the reproducibility of histological assessments.

This situation demands the exploration and validation of complementary methodologies that offer objective and quantifiable evaluation. The search for tools that reduce human bias and provide reliable data is crucial to optimize graft selection and improve renal transplant outcomes [23].

In the search for a more objective and precise method for evaluating renal organ viability in the transplantation process, bioimpedance spectroscopy technology emerges as a promising alternative. This technique, already established for the evaluation of biological tissues, can provide valuable information about physiological and morphological conditions [24,25,26]. The temporal dynamics of bioimpedance measurements have been noted as a sensitive indicator of structural and morphological changes [27,28]. It has been applied in the objective evaluation of tissue and cell culture viability [29,30], organ ischemia monitoring [31,32], discrimination between healthy and tumor tissue [26,33], and body composition assessment in renal transplant patients [34,35]. Although there are studies exploring its use in evaluating kidney viability for liver transplantation [36], its application in renal transplantation remains unexplored. Furthermore, detailed knowledge of the conductive and bioimpedance properties of the living kidney is limited, despite evidence suggesting a direct correlation between the temporal variability of bioimpedance and the evolution of ischemia [37]. Therefore, research in this field presents an opportunity to improve renal graft viability assessment protocols.

Bioimpedance spectroscopy, although widely recognized for its utility in assessing post-transplant nutritional status due to its association with graft viability [38], faces significant challenges in its intraoperative application for evaluating organ suitability for transplantation. The scientific literature presents preliminary results, predominantly based on animal organs such as rodents [39], lagomorphs [40], and porcine models [32]. These studies, often focused on the time-dependent monitoring of ischemia effects on bioimpedance parameters, suffer from limitations in the physiological interpretation of bioimpedance variations. Similarly, the application of bioimpedance in ex vivo organs, aimed at discriminating between normal and pathological tissues, has been explored in studies with human organs [41,42]. However, these analyses are typically performed statically, without considering the influence of ischemia and cell death, and are limited to the study of tissue samples, without addressing the complexity of the entire organ. Consequently, there is a clear need to develop methodologies that allow for the dynamic analysis of bioimpedance parameters and their correlation with underlying physiological processes.

In the field of bioimpedance spectroscopy, modeling emerges as a fundamental tool for extracting relevant information from the analyzed frequency spectrum. The Cole model, widely adopted in this context, stands out for its simplicity, which facilitates rapid fitting suitable for real-time monitoring, as well as for the physical and physiological relevance of its parameters, which allow for subsequent clinical study and analysis [43,44,45]. However, the repeatability of Cole parameters presents an unsolved challenge, largely attributable to the uncertainty derived from the inherent limitations of the model [43]. This problem underscores the need to explore alternative models or modifications of the Cole model that improve the accuracy and repeatability of the estimates.

### 1.1. Hypothesis and Objectives

In this context, a series of hypotheses are raised to be investigated:

- Can a bioimpedance measurement device be developed and adapted to the surgical environment to provide real-time information on the ischemic status of the kidney organ during the transplant process?
- Can measurement schemes and new bioimpedance models be defined to improve the problem of repeatability of bioimpedance measurements in organs?

To answer these questions, this paper sets out the following objectives:

- The main objective is to present an intelligent bioimpedance spectroscopy device for assessing kidney viability during renal transplantation. To address this objective, the starting point is a multifrequency bioimpedance device patented by the Biomedical Engineering Group of the University of Seville [46], which has been validated and used in various clinical applications [47,48]. This work focuses on the research, development, and evaluation of instrumentation adapted to the renal transplant scenario and its approximation to the surgical environment, emphasizing the need for sterilization. Some preliminary and partial results on the researched aspects were presented in [49,50,51]. They are expanded on and discussed in this paper from a more integrated perspective. Other novelties of this work are related to the sensing stage and a modification of the four-electrode measurement scheme to provide greater accuracy and repeatability in bioimpedance estimations. On the other hand, the situation generated by the COVID-19 pandemic raised the need to adapt the device so that measurements could be performed autonomously by clinical staff without technical personnel intervention, leading to modifications in the user interface and data storage compared to the starting device [47].
- Another objective of the work is the proposal of a new Cole model, designed to address the problem of parameter repeatability in bioimpedance measurements. This model is distinguished by its ability to offer accurate and repeatable estimations in short-duration measurements, ranging from 1 to 5 min, and facilitate the analysis of long-term parametric variations, where samples are separated by intervals of 1 h or more. This duality allows for a more complete and reliable evaluation of bioimpedance dynamics at different time scales, overcoming the limitations of the conventional models.

### 1.2. Structure of the Work

This paper is structured as follows: after this introduction, in Section 2, the materials and methods used in the work are outlined, including the proposed bioimpedance models; in Section 3.1, the design, development, adaptations, and improvements made to the bioimpedance device are described; in Section 3.2, the results of a feasibility study of the device to measure renal bioimpedance are presented; in Section 3.3 the results of therepeatability of the measurements are indicated, both in the short and long term; in Section 3.4, the results of the analysis of the new proposed models are shown; in Section 3.5, the evolution of the bioimpedance parameters as the ischemia time increases is analyzed; and Section 4 and Section 5 are related to the discussion and conclusions of the work. Finally, Appendix A describes the protocol developed for performing measurements in the operating room with the device, and Appendix B describes the user manual that has been developed to facilitate the use of the device by clinical staff.

## 2. Materials and Methods

### 2.1. User-Centered Design Methodology

The adaptation of the bioimpedance spectroscopy device to the renal transplant environment has been based on a user-centered design methodology, adopting a functional modularity approach that facilitates the integration of emerging technologies and functionalities, and adhering to recognized standards. A series of semi-structured interviews were conducted to identify needs, gather opinions, and collect improvements suggested by the clinical team regarding the user interface, data storage, and measurement procedure. An iterative development cycle, carried out in parallel with the evaluation, was employed, which involved the evolution of the user interface and the measurement device through several prototypes to develop the final device.

Although the work presents final development results, the iterative user-centered design methodology has resulted in several prototypes of devices, probes, protocols, and interfaces. To summarize, the following optimizations from the previous device [47] have been performed:

- A series of improvements with the purpose of adapting its functionality for measurements in kidneys and in a surgical environment. The previous prototype required the assistance of specialized technical personnel to carry out the measurements, which limited its autonomy and hindered its implementation in the surgical environment, especially during the COVID-19 pandemic. The need to minimize the presence of additional personnel in the operating room, to reduce the risk of contagion, and ensure compliance with safety protocols made it essential to develop a system that would allow the clinical team to perform measurements autonomously and safely. The transition to autonomous use of the measurement device by the clinical team required modifications to both the hardware and the measurement protocols.
- The improvements provided by the process have resulted in an optimization of the current generation hardware and the input stage of the instrumentation amplifier.
- The measurement procedure has undergone changes due to optimizations made to the measurement scheme to improve repeatability, from localized measurements to longitudinal measurements.
- Processing algorithms for estimating bioimpedance parameters that synthesize the organ’s bioelectrical behavior have resulted in several models with the aim of minimizing fitting error and improving measurement repeatability.
- The need to sterilize the electrodes has led to a protocol for using the device that had to be approved by the Central Sterilization Unit of the Preventive Medicine and Public Health Service at the Virgen del Rocío University Hospital in Seville.
- The greatest variability in prototypes was established in the design of the probes, until a suitable solution was found for safe use in the operating room.
- A physical integration of the control device and the measurement device described in [48] was carried out. This integration resulted in a unique and compact housing, which houses both the data acquisition and processing components, as well as the user interface.
- A standardized and easy-to-follow measurement protocol was established to facilitate data management during transplantation and the recording of data of interest.
- User interface has been designed according to the needs of the clinical staff, allowing the visualization of results and the control of the device efficiently and safely. Usability and accessibility of the user interface have been established through different means (graphic, acoustic, and touch).

### 2.2. Technical and Clinical Requirements

The conception of the device was based on a series of fundamental design requirements, aimed at ensuring its functionality and safety in the clinical environment. Among these requirements, the following stand out:

- A compact and sterilizable design, indispensable for its use in the operating room.
- Immunity to electromagnetic interference, crucial for measurement stability.
- Portability, which facilitates its transport and handling.
- Measurement accuracy, to ensure the reliability of the results.
- Biocompatibility, which ensures the absence of adverse reactions in the patient.
- Cost minimization, in order to expand its accessibility.

These requirements were meticulously considered and agreed upon with the clinical team throughout the development process, with the aim of creating a device that met the demands of the surgical environment and offered accurate and reliable results.

### 2.3. Reference Pattern

In order to preliminarily validate the instrumentation stage of the device, a reference circuit was designed capable of simulating various bioimpedance values within a range representative of measurements obtained in human kidneys. The use of a reference standard/pattern is necessary to validate the device for bioimpedance measurements. Since the bioimpedance values of organs and biological tissues are unknown a priori, it is necessary to establish an equivalent electrical circuit that exhibits impedance values and behavior comparable to those observed in kidney tissue. The determination of this range was based on experimental results previously obtained in kidney studies, which allowed for the establishment of a realistic and relevant reference pattern for device validation. The use of this circuit pattern allowed the evaluation of the accuracy of the bioimpedance measurements generated by the device, ensuring its correct functioning before its application in clinical studies.

**Figure 1:**
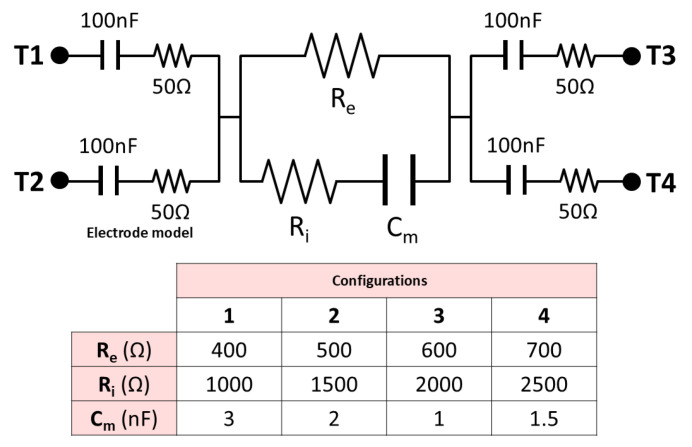
Schematic of the circuit pattern designed for device validation.

The schematic representation of the reference circuit, as well as the various configurations it supports, are illustrated in Figure 1. This circuit incorporates an electrode model, composed of a capacitance of 100 nF and a 50 ohm resistor arranged in series, to more accurately simulate real measurement conditions. Terminals T1 and T3 are intended for current injection, while terminals T2 and T4 are used for voltage measurement. The selection of component values was based on the researchers’ accumulated experience. For the evaluation of the device’s accuracy, the error in the measurements is used, considering in this case the relative error. In this case, it is defined as the Euclidean distance in the bioimpedance complex plane normalized by the expected absolute value of the bioimpedance, and expressed as a percentage. A total of 22 frequencies were analyzed, logarithmically distributed in the range between 5 kHz and 1 MHz.

### 2.4. Types of Measurements

To study the feasibility of bioimpedance technology in renal organs, two types of measurements have been performed:

- Bioimpedance measurements in pig kidneys: In order to evaluate the repeatability and reliability of bioimpedance measurements, an experimental study was conducted using kidneys obtained from pigs slaughtered in abattoirs intended for human consumption. The selection of this animal species was based on the marked anatomical similarity between porcine and human kidneys, validated by comparative measurements in human nephrectomy samples (see Section 3.2), which ensures the relevance of the results obtained for clinical application in humans. In addition, measurements in pig kidneys have allowed for the analysis of the evolution of ischemia and cell death in renal tissue, avoiding the use of human samples. The measurement protocol in pig kidneys is described below: Approximately 20 min after the animal’s sacrifice, the organ was extracted, initiating the sequence of measurements. To recreate the conditions prevalent in the surgical environment, porcine kidneys were immersed in an organ preservation solution (CELSIOR^®^ from IGL, Lissieu, France), both at the beginning of the experiment and during intervals between measurements. The temperature of the organs was maintained in a range of 2 °C to 8 °C using ice in a refrigerated container, thus simulating the storage and preservation conditions common in an operating room. Immediately before each measurement, the organs were removed from the preservation solution, excess moisture was carefully removed using sterile gauze, and they were placed on an insulating surface. This procedure was carried out to ensure the accuracy of the measurements and avoid the influence of external factors, such as moisture or the conductivity of the support surface.
- Bioimpedance measurements in human kidneys: In order to validate the applicability of the device in measurements on human kidneys, a second evaluation was carried out using kidneys extracted from patients undergoing complete nephrectomy. This study was approved by the Ethics Committee of the Virgen del Rocío University Hospital of Seville, ensuring compliance with ethical and legal principles in research with human samples. In this case, only several measurements were considered at a specific point in time to analyze measurement repeatability and confirm the suitability of using pig kidneys as a model for the human kidney due to similarity in results.

### 2.5. Study of Repeatability

An exhaustive study was conducted to evaluate the repeatability of the measurements, in order to determine the reliability and robustness of the measurement system under different experimental conditions. To quantify the stability and evolution of the bioimpedance model parameters, an analysis based on the percentage standard deviation relative to the absolute value was performed. This analysis was carried out considering two distinct experimental scenarios, for which an experimental protocol defining the sequencing of measurements was designed:

- Short-term repeatability assessment: Consecutive measurements, comprised within a global time interval of less than 5 min, were analyzed. Between each measurement, the probe was removed from the kidney, allowing for the evaluation of the influence of slight variations in electrode position on the contact area. This scenario provided information on the repeatability of measurements under short-term stability conditions.
- Long-term evolution assessment: Measurements spaced over time were analyzed to record the evolution of parameters as the ischemia process progressed. This scenario allowed for the evaluation of the models’ ability to capture the dynamic changes in bioimpedance over an extended period.

This dual approach allowed for a comprehensive view of the stability and evolution of bioimpedance model parameters under different experimental conditions, and during the evolution of the ischemia process.

The evaluation of measurement repeatability was based on comparing the standard deviation observed in the long-term evolution study with the standard deviation obtained in short-term successive measurements. Since there is no prior evidence of whether the range of variation in bioimpedance parameters in successive measurements is indicative of lower or greater repeatability, its significance has been established in relation to the full range of variation in the parameters in a study of evolution over time. In this context, it was defined as the reverse repeatability parameter as the ratio between the standard deviation of successive measurements and the standard deviation of the temporal evolution study, expressed as a percentage. In other words, the smaller the reverse repeatability parameter, the greater the repeatability of the measurements. This favors long-term bioimpedance measurements providing sufficient variability to allow for the distinction of parameter evolution over time. This requirement was considered essential to ensure the device’s ability to detect changes in renal tissue, which is particularly relevant for its application in graft viability assessment in the context of transplantation.

### 2.6. Study of New Bioimpedance Models

The variability found in bioimpedance parameters in pig kidney measurements revealed the need to explore bioimpedance models alternative to the Cole model ($Z_{0}$) [43,44,45]. This search is based on the aspiration to minimize estimation error (accuracy as defined in Section 2.3), through a more precise fit to bioimpedance data, and to address the problem of model parameter repeatability in consecutive measurements. The ultimate goal was to mitigate the impact of possible uncertainty factors that may compromise measurement reliability. In order to optimize the Cole model for renal bioimpedance analysis, two possible modifications were explored:

- Cole model with parasitic capacitance ($Z_{1}$), which incorporates a parasitic shunt capacitance ($C_{P}$) with the kidney’s bioimpedance. This inclusion is justified by the need to model the capacitive effects that may arise due to the presence of conductive elements in the probes and electrodes used in the measurement, as well as the capacitive effects derived from the influence of external ground. Parasitic capacitance represents an additional impedance that can affect measurement accuracy, especially at high frequencies. By incorporating this capacitance into the model, the aim is to improve the representation of the total system impedance and, therefore, obtain more accurate estimates of kidney bioimpedance.
- Cole model with frequency-dependent membrane capacitance ($Z_{2}$): In this work, a new model has been proposed in which membrane capacitance increases linearly with frequency. This modification sought to improve the model’s ability to represent the complex interaction between electrical current and the cellular structures of renal tissue.

The mathematical expressions that define both the conventional Cole model ($Z_{0}$) and the proposed variants, $Z_{1}$ and $Z_{2}$, which aim to improve the representation of renal bioimpedance, are presented below:

(1) $$Z_{C}=R_{\infty }+\frac{R_{0}-R_{\infty }}{1+{\left(j2\pi f\left(R_{E}+R_{I}\right)C_{m}\right)}^{\alpha }}$$

(2) $$Z_{0}=Z_{C}e^{-j2\pi fT_{D}}$$

(3) $$Z_{P}=\frac{1}{j2\pi fC_{P}}$$

(4) $$Z_{1}=\frac{Z_{C}\cdot Z_{P}}{Z_{C}+Z_{P}}e^{-j2\pi fT_{D}}$$

(5) $$Z_{M}=R_{\infty }+\frac{R_{0}-R_{\infty }}{1+{\left(j2\pi f\left(R_{E}+R_{I}\right)\left(A\cdot f+C_{m,LF}\right)\right)}^{\alpha }}$$

(6) $$Z_{2}=Z_{M}e^{-j2\pi fT_{D}}$$

The equations defining the bioimpedance models include several parameters with specific physical and physiological meanings:

- $R_{0}$ (Resistance at zero frequency, in ohms): It represents the resistance that tissue offers to the passage of a direct electrical current. In the biological context, it is mainly associated with the resistance of the extracellular fluid.
- $R_{\infty }$ (Resistance at infinite frequency, in ohms): It represents the tissue’s resistance when the frequency tends to infinity. In this case, the electrical current passes through both the extracellular and intracellular fluids, so $R_{\infty }$ reflects the total resistance of the tissue.
- $R_{I}$ (Intracellular resistance, in ohms): It is calculated from $R_{\infty }$ and $R_{0}$ ($R_{I}=R_{\infty }R_{0}/\left(R_{0}-R_{\infty }\right)$), and represents the resistance offered by intracellular components to the passage of electrical current.
- $R_{E}$ (Extracellular resistance, in ohms): It corresponds to $R_{0}$, and represents the resistance offered by the extracellular fluid to the passage of electrical current.
- *f* (Frequency, in Hz): It is the frequency of the electrical current used in the bioimpedance measurement. The tissue’s response varies depending on the frequency, which allows for obtaining information about its different components.
- $C_{m}$ (Membrane capacitance, in Farads): It represents the electrical capacitance of cell membranes. Its value is associated with the insulating behavior of cell membranes.
- $C_{m,LF}$ (Membrane capacitance at low frequency, in Farads): It is the value of the membrane capacitance at low frequencies of the $Z_{2}$ model proposed in this work.
- *A* (Modification factor of $C_{m}$, in Farads per Hz): It is a parameter that models the dependence of membrane capacitance on frequency.
- $T_{D}$ (Phase delay, in seconds): It represents the delay in the phase of the electrical current due to the length of the cables and the characteristics of the hardware used in the measurement. This parameter is important to correct possible errors in bioimpedance measurements.

The determination of the parameters of the mathematical models, used to describe bioimpedance measurements, was carried out by implementing genetic algorithms. A population size of 5000 and 12 generations were used to minimize mean squared error and reduce total processing time. At each iteration, the top 20% of genes remained fixed. For the remaining top 30% of genes the probability of mutation varied linearly from 0.07% to 21%, and the rest were completely altered with a new randomized parameter space. This optimization process was performed in the MATLAB programming environment (version R2023a), selecting the parameters that minimized the mean squared error between the experimental data and the values estimated by the models.

In the comparative study of models $Z_{0}$, $Z_{1}$ and $Z_{2}$, the standard deviation of the measurements was used against the bioimpedances resulting from the corresponding model that offered the best fit. This evaluation was performed on bioimpedance measurements obtained from pig kidneys.

## 3. Results

### 3.1. Design and Development of the Bioimpedance Prototype

**Figure 2:**
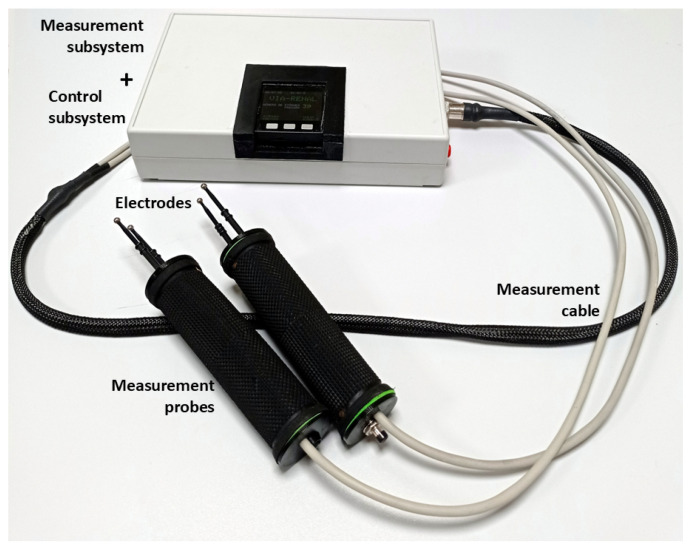
Bioimpedance system for the evaluation of renal viability and pathophysiological status.

**Figure 3:**
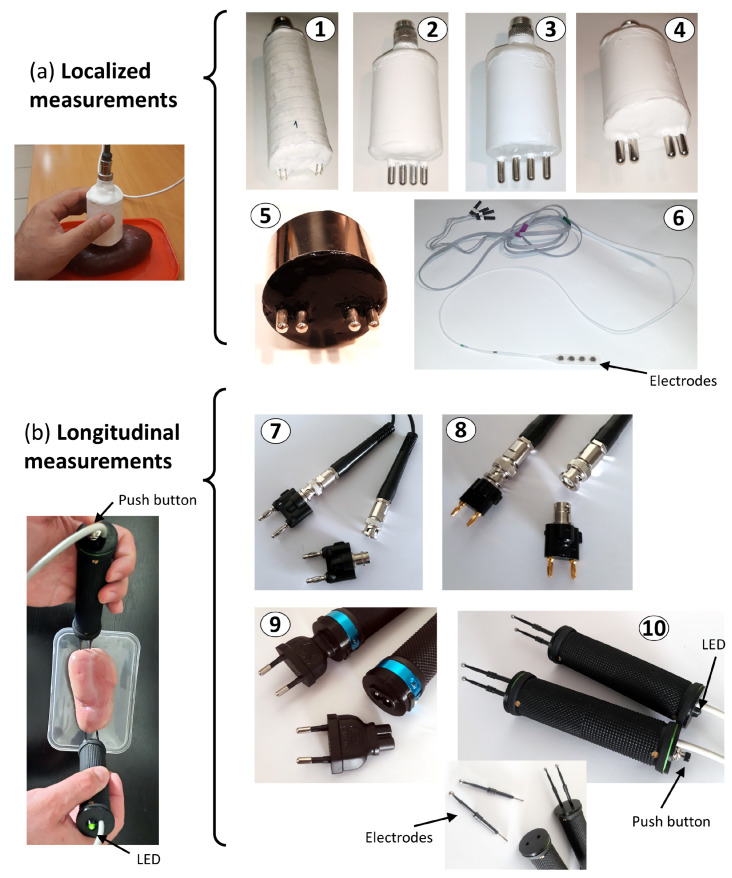
Probes developed for kidney measurements: (**a**) Localized measurements (probes number 1 to 6). (**b**) Longitudinal measurements (probes number 7 to 10). The figure also describes the positioning of the electrodes on a pig kidney in both schemes.

**Figure 4:**
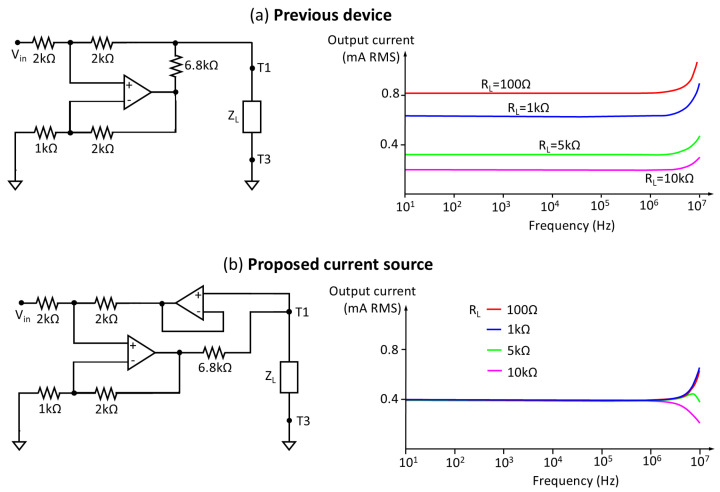
Comparative schematics of the current source and simulated injected current as a function of load impedance: (**a**) previous device configuration [47]; (**b**) improved current source proposed in this work.

**Figure 5:**
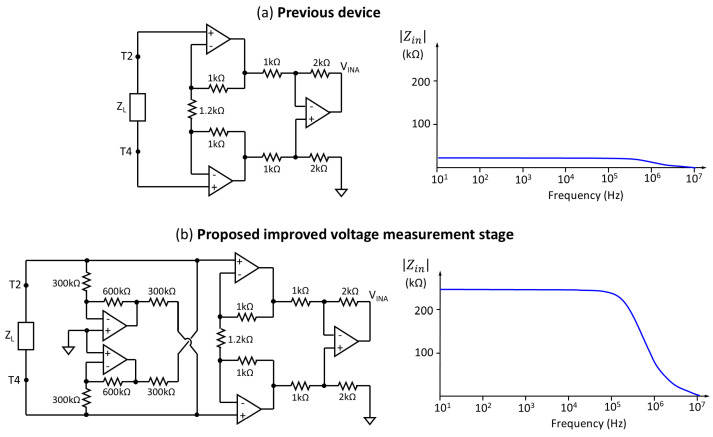
Comparative schematics of the voltage measurement stage and simulated input resistance as a function of frequency: (**a**) previous device INA [47]; (**b**) improved voltage measurement stage proposed in this work.

**Figure A2:**
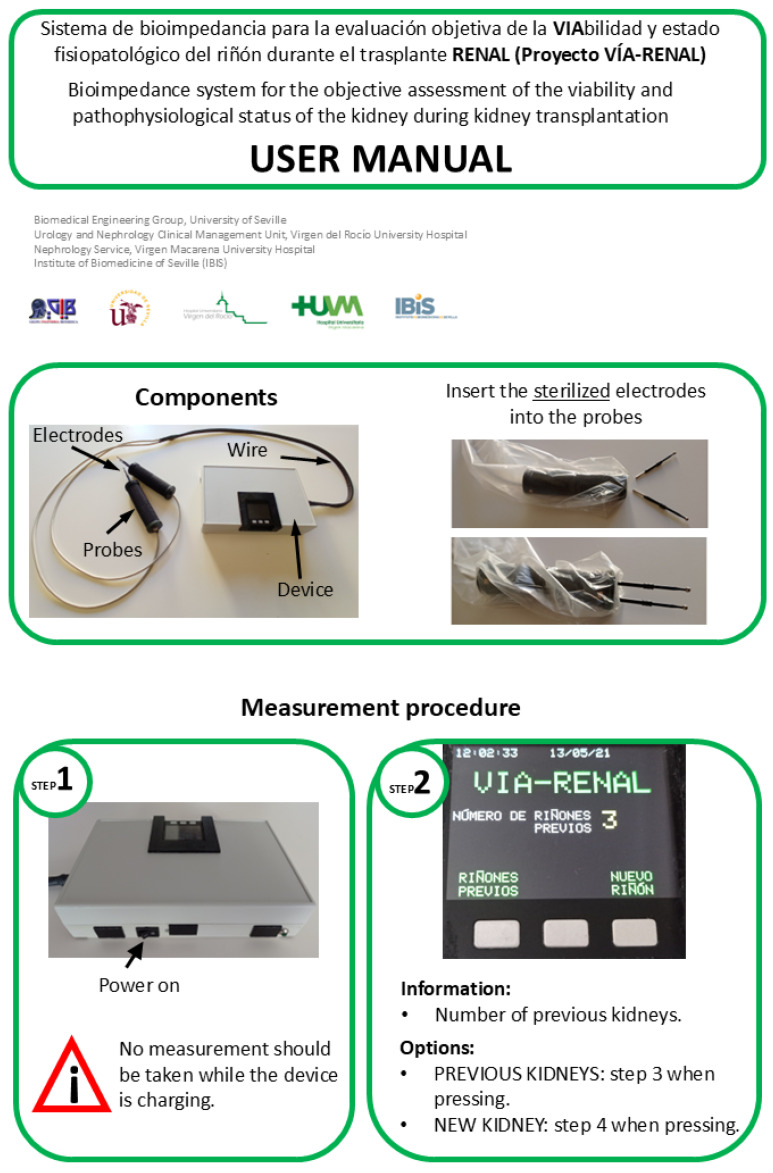
English translation of the user manual (page 1).

**Figure A3:**
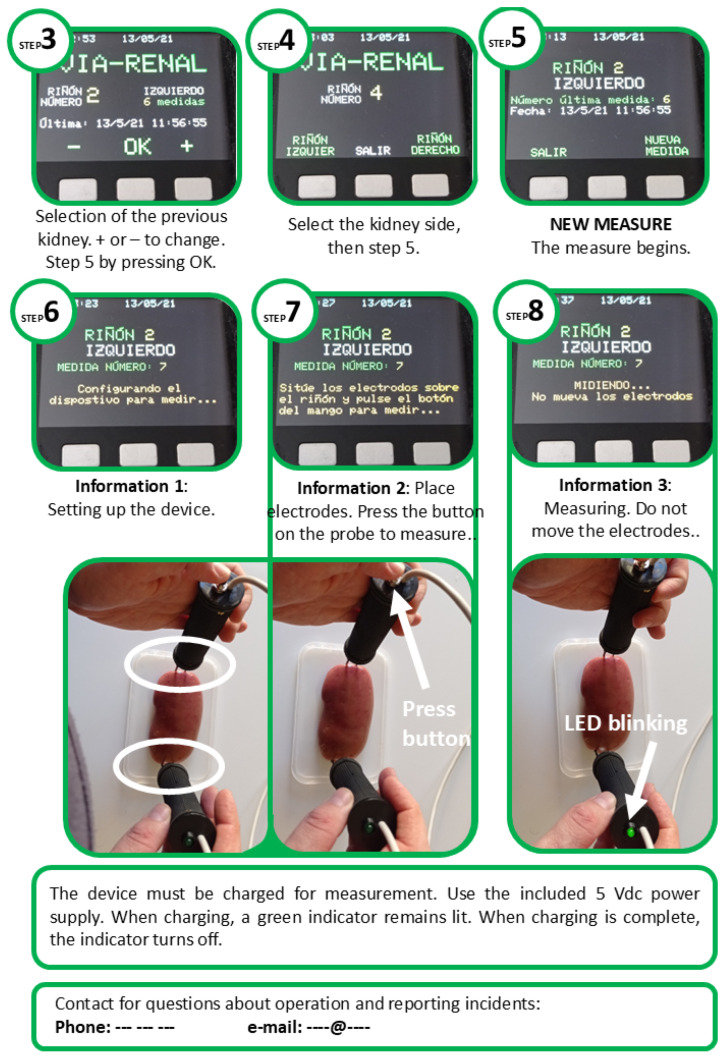
English translation of the user manual (page 2).

The resulting bioimpedance measurement system is characterized by a modular design architecture, which facilitates the integration of new technologies and functionalities, as well as its adaptation to different clinical scenarios (see Figure 2):

- Measurement probe: In order to guarantee the safety and sterility of the measurement environment, a configuration has been adopted that incorporates the electrodes into two ergonomic probes, designed to facilitate their handling by the operator. This arrangement allows for the individual extraction of the electrodes for sterilization, minimizing the risk of contamination and ensuring asepsis during the measurement procedure. The optimal design of the probe, both in its location and assembly, was the subject of an exhaustive analysis by the clinical and technical team. This collaborative process resulted in the identification of a solution that allows for obtaining accurate and reliable measurements, while facilitating the usability and handling of the probe by medical personnel during the kidney transplant procedure. During the design and optimization process of the probe, an exhaustive analysis of various factors that could influence the performance of the measurement system was carried out. The distance, shape, and dimensions of the probe and electrodes were thoroughly investigated, in order to determine the optimal configuration for obtaining accurate and reliable measurements. Likewise, mechanical and physical aspects of the probe, such as weight, measurement pressure, and grip area, were analyzed in depth, with the aim of ensuring its ergonomics and facilitating its handling during the surgical procedure. Figure 3 illustrates a selection of the probes evaluated during the design process, with two main types being distinguished:
  (a) Probes for localized measurements: These probes were designed to measure in a specific area of the kidney, with fixed distances in the separation between the electrodes. Probe 1 was a first approximation, but was discarded for having sharp elements that could damage the organ. Probes 2, 3, and 4 had a rounded shape at their ends, more suitable for their placement on the organ, with different configurations of distances between the electrodes, but these probes were also discarded for not being suitable for heat sterilization. Probe 5 implemented the most sensitive option of the previous probes, thanks to the greater distance between the electrodes intended for voltage measurement, and was specifically designed to be heat-sterilized by means of a housing composed of stainless steel and silicone suitable for withstanding high temperatures. Probe 6 was based on disposable or sterilizable commercial electrodes, specifically intraoperative subdural neurophysiology electrodes (MS04R-IP10X-0JH from Ad-Tech, Ashburn, VA, USA). In all the probes for localized measurements, current injection was carried out through the outer electrodes, while voltage measurement was established through the inner electrodes.
  (b) Probes for longitudinal measurements: Figure 3b shows an illustrative image of the electrode arrangement for longitudinal measurements in the kidney. The current injector electrodes are positioned at opposite ends of the organ, while the voltage measurement electrodes are located adjacent and close to the injector electrodes, also at the ends of the kidney. This configuration allows a uniform distribution of current through the renal tissue and the measurement of the impedance of the entire kidney, which is fundamental to obtain bioimpedance measurements representative of the physiological state of the organ. As will be seen in Section 3.3, the adopted measurement configuration demonstrated superiority in terms of repeatability compared to localized measurements in specific regions of the organ. This global measurement scheme minimizes the influence of local variations in the bioimpedance of the renal tissue, thus providing more consistent and reliable results. Different options were evaluated in which the probe incorporated a removable and sterilizable element, which could be incorporated into the probe’s handle by means of a connector. Probes 7 and 8 showed two solutions based on BNC connectors, which were discarded due to the small size of the electrode tips that could cause damage to the kidney. Probe 9 provided another option with a more rounded tip based on an electrical plug. Probe 10 demonstrated the finally adopted solution, based on commercial electrosurgery electrodes (F4068 from FIAB, Vicchio, Italy).

- Electrodes: A four-electrode configuration has been implemented instead of the standard two-electrode configuration, with the aim of removing the influence of the electrodes’ own impedance on the measurements. The impedance of the electrodes, which can vary significantly depending on their material and geometry, as well as the contact with the tissue, introduce sources of error in bioimpedance measurements. By using a four-electrode configuration, current injection is separated from voltage measurement, thus eliminating the influence of electrode impedance on tissue bioimpedance measurement. As previously mentioned in the description of the measurement probe, and as can also be seen in Figure 3, different electrodes were studied and analyzed. However, the exhaustive investigation of sterilization procedures led to the selection of a design alternative based on electrodes approved for sanitary use, with CE certification, and compatible with high-temperature autoclave sterilization. After a detailed evaluation of the different options, the FIAB F4068 commercial electrosurgery electrodes were chosen, and they were integrated into a particular solution of probes for longitudinal measurements. In this configuration, the electrodes can be easily inserted and removed individually thanks to a connector integrated inside the probe. These electrodes, characterized by their smoothed spherical shape with a diameter of 4 mm at the contact end with the renal tissue, minimize the risk of iatrogenic damage during the measurement process. The atraumatic morphology of these electrodes is crucial to ensure patient safety and renal graft integrity during bioimpedance evaluation. Figure 3b illustrates the details of probe 10 developed for its application in the surgical environment, highlighting the characteristics of the electrodes and their integration into the probe assembly. In addition, its design allows for high-temperature autoclave sterilization, which facilitates its reuse and reduces the risk of nosocomial infections.
- Measurement cable: The system design incorporates a cable that connects the probe to the measurement subsystem, with the purpose of optimizing the ergonomics of the procedure and facilitating the sterilization of the components. The measurement cable has a length of 180 cm to allow physical separation of the measurement area from the device. In addition, it is protected with electromagnetic shielding to mitigate the influence of electromagnetic noise in the clinical environment, where the presence of electronic equipment and interference sources is common. This separation between the probe and the main device allows for greater freedom of movement during measurement, which is especially useful in surgical environments where space and organization are critical. To ensure the asepsis of the measurement procedure, the use of a sterile and disposable cover has been implemented that covers both the measurement probes and the connection cable, with sufficient length to allow comfortable handling. An 8-pin connector with a threaded/bayonet locking mechanism is used to ensure a firm and stable connection, preventing accidental disconnections during the measurement procedure. The design of the connectors facilitates their installation and handling, which is crucial in surgical environments where speed and precision are essential. Of the 8 pins, 4 are used for bioimpedance measurements, and the rest are used for shielding and handling a push button and an indicator LED on the probes, as will be described later.
- Measurement subsystem: The measurement subsystem is responsible for the acquisition and processing of signals and the estimation of parameters that allow the evaluation of the electrical properties of renal tissue. This device is responsible for generating the electrical current, measuring the resulting voltage, and calculating the complex impedance of the tissue. The choice of the multifrequency spectroscopy technique, instead of the standard single-frequency method, is based on the need to increase the sensitivity and specificity of the measurement system by enabling the differentiation between intracellular and extracellular compartments. This selective analysis capability facilitates the implementation of a measurement normalization process, an essential aspect in the proposed system. Normalization allows correcting the variations inherent to the individual characteristics of the kidneys, thus ensuring that accurate and comparable measurements are obtained between different organs and patients. At each frequency, estimates of both the modulus and phase of the complex impedance occur. This wide frequency coverage allows for detailed characterization of the electrical properties of the renal tissue, providing valuable information about its composition and structure. Based on the system described in [47], the main elements of the measurement subsystem that have been modified with respect to the previous prototype are described below:
  (a) Regarding the device’s power supply, the previous prototype integrated the TPS65133 DC-DC converter from Texas Instruments (Dallas, TX, USA), designed to supply the ±5 V voltage levels required by the analog sensing stage [47]. However, this converter exhibited susceptibility to start-up failures in the case of the initial current demands exceeding a predefined threshold. The mitigation of this problem was achieved through the implementation of a soft start procedure, orchestrated through a manual activation sequence of three switches. In the current prototype, the incorporation of two load distribution switches (TPS22918DBVT from Texas Instruments, Dallas, TX, USA), controlled by software, has been chosen. This modification allows the device’s power-on management through a single button, thus simplifying autonomous operation, eliminating the possibility of operational errors derived from incorrect power-on sequences, and substantially improving the reliability and usability of the device.
  (b) The generation of sinusoidal signals, whose frequency is configurable, has been implemented using the Analog Devices AD9854 DDS (Wilmington, MA, USA) integrated circuit, which allows generating sinusoidal signals with high precision and stability over a wide frequency range.
  (c) The conversion of the sinusoidal voltage generated by the AD9854 DDS into a constant amplitude current (0.4 mA RMS) is performed by an improved Howland current pump, based on the previous design described in [47]. The amplitude of the current used is well below the international safety limits established for bioimpedance applications [52], which ensures the safety of the organ during the measurement procedure. Compared to the previous device [47], an improvement is proposed in the electrical current generation stage. This modification has the main objective of making the injected current independent of the load impedance value, which in this context corresponds to the bioimpedance of the kidney. The dependence of the injected current on the load impedance can introduce significant errors in bioimpedance measurements, since variations in the renal tissue impedance (due to factors such as hydration, temperature, or physiological state) would directly affect the magnitude of the current. By improving the stability of the constant current source, it is ensured that the injected current remains stable regardless of fluctuations in the kidney impedance. This results in more accurate and reliable measurements, which is fundamental for the objective evaluation of renal graft viability. The independence of the injected current from the load impedance also allows for greater repeatability of measurements, which is crucial for monitoring subtle changes in the bioelectrical properties of renal tissue during transplantation. Figure 4 illustrates the comparative design of the current source and the quantifiable improvements obtained. Unlike the preliminary prototype, where calibration compensated for load variations, the new current generation stage ensures more precise measurement and strict control of the electrical current.
  (d) An instrumentation amplifier (INA) based on operational amplifiers is used for voltage measurement. The gain of this amplifier has been adjusted to provide the largest dynamic range in the impedances obtained in a kidney, and ensuring to avoid signal saturation in all possible cases. A modification of the instrumentation amplifier design has been implemented, schematically represented in Figure 5b, which increases the input impedance by an order of magnitude (factor of 10). This optimization is crucial for the accuracy of the voltage measurement, since the high input impedance minimizes the influence of the electrode impedance on the obtained estimates (see Figure 5b). Reducing the effects of load impedance is essential to ensure the reliability of bioimpedance measurements, especially in biological tissues that exhibit variable intrinsic impedance. The implementation of this circuit modification also contributes to obtaining more accurate and representative measurements of the bioelectrical properties of renal tissue.
  (e) The measurement subsystem includes a first processing of the acquired signals to determine the modulus and phase of the bioimpedance at each of the frequencies, applying the quadrature measurement technique described in [47]. The processing results are sent through a formatted data structure to the control subsystem via a wired serial data interface.

- Control subsystem: The control architecture was implemented using an M5Stack module (M5Stack Technology, Shenzhen, China), which was physically integrated together with the measurement subsystem in the same housing and measurement device. This convergence of functionalities in a single compact unit eliminated the dependence on an external device, such as a smartphone [47] or laptop [48], thus optimizing the portability and operational efficiency of the system. Based on the previous system, the main elements of the control subsystem that have been modified with respect to the previous prototype are described below:
  (a) Data storage: The persistence of acquired data is guaranteed by their storage in files hosted on an SD memory card. This external storage strategy minimizes the vulnerability of information to overwrite events that could compromise the integrity of the device’s internal memory. The use of wireless communications is restricted exclusively to external debugging procedures. Patient data were anonymized to ensure information security and privacy.
  (b) Graphical user interface: The device’s human-machine interface is materialized through the LCD screen and three push buttons integrated into the M5Stack module, which allow direct interaction with the control software and navigation through specific menus. This graphical interface facilitates the execution of a standardized measurement protocol, designed in collaboration with the clinical team, and provides the following essential functionalities: (1) assignment of a unique numerical identifier to each renal graft; (2) specification of the graft’s laterality (right/left) for correct anatomical identification; (3) sequential recording of the measurement number performed on a specific graft, allowing monitoring of the evolution of bioelectrical properties; (4) selection of the target graft for the acquisition of new measurements, which enables interleaved operation of the device with multiple renal grafts. This last functionality is particularly useful in clinical scenarios where simultaneous evaluation of several grafts is required. To facilitate the handling of this interface, a user manual was developed and training sessions were conducted for the clinical team. The English translated version of the user manual, which highlights the different stages of the measurement process, is included in Figure A2 and Figure A3 of Appendix B.
  (c) Acoustic interface: The human-machine interface is complemented by an acoustic feedback system, designed to provide distinctive auditory signals that indicate the beginning and end of the bioimpedance measurement process. Additionally, this acoustic signaling system is used to communicate to the user relevant actions related to interaction with the graphical interface, such as confirmation of menu option selection or error notification. The implementation of this sensory feedback modality contributes to improving the usability of the device, especially in clinical environments where the user’s visual attention may be limited. The combination of visual and auditory signals ensures effective communication between the device and the user, facilitating the execution of measurement procedures and minimizing the possibility of operational errors.
  (d) Touch interface: The measurement probe incorporates an activation and signaling mechanism to optimize the data acquisition process. A push button, strategically located on one of the probe handles, allows the user to initiate the bioimpedance measurement once the electrodes have been correctly positioned on the renal organ. Additionally, an LED indicator light, integrated into the opposite handle, provides a visual signal of the measurement status. This LED emits an intermittent light signal during the 8 s that the data acquisition process lasts, alerting the user to the need to keep the probe in a static position. This functionality is crucial to minimize motion artifacts and ensure the accuracy and repeatability of bioimpedance measurements. The integration of these control and signaling elements into the measurement probe facilitates the operation of the device by clinical personnel, contributing to obtaining reliable data and optimizing workflow in the surgical environment, since all control is derived from the measurement probes at the moment the electrodes are placed on the renal organ for measurement.
  (e) Real-time clock: Each bioimpedance measurement is associated with a precise timestamp (date and time) thanks to the implementation of a real-time clock with its own battery based on the DS3231 module (Maxim Integrated, San Jose, CA, USA). This functionality facilitates the chronological correlation of bioimpedance data with relevant clinical information of the renal transplant during retrospective analysis and evolution studies. Considering that measurements can be performed simultaneously on several kidneys, and that, according to the measurement protocol, measurements are planned to be taken at two moments (after extraction and before implantation), adequate traceability of the measurements is ensured thanks to the real-time clock that assigns to each measurement the time at which it was carried out.

Given the high number of alternatives studied (different probes, different cables and lengths, different gain conditions in voltage measurement, etc.), and since each alternative requires a different system calibration, the semi-automatic self-calibration procedure described in [47] was used. Once the device is calibrated, the acquisition of a complete multifrequency bioimpedance spectrum is performed in a span of eight seconds. This optimized measurement time enables obtaining information about the electrical properties of renal tissue quickly and efficiently, minimizing the disruption of the clinical workflow.

### 3.2. Feasibility of Using the Device for the Study of Renal Bioimpedance

To validate the accuracy and reliability of the device as a bioimpedance measurement instrument, after calibrating the device, an exhaustive evaluation of all the circuit pattern configurations for each considered design alternative (probe, cable length, etc.) was carried out. The analysis of the error associated with the estimates revealed that, in all the cases, the error remained below the 1% threshold. The consistency of the estimates, with minimal error, supports the robustness of the design and the effectiveness of the implemented calibration procedures. The use of a bioimpedance pattern is necessary because the bioimpedance of a kidney is unknown and cannot be used as a reference in validation.

**Figure 6:**
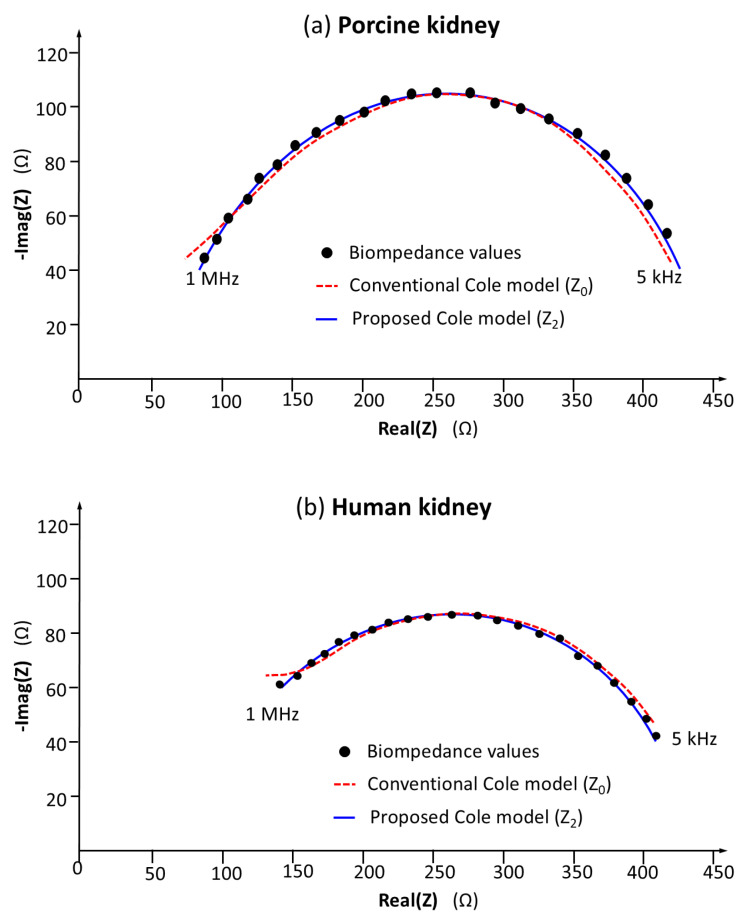
Example of bioimpedance measurement performed with probe 10: (**a**) porcine kidney bioimpedance values; (**b**) human kidney bioimpedance values. To show the agreement between the bioimpedance values and the Cole models, the resulting values of $Z_{0}$ and $Z_{2}$ models are also included.

Following the technical validation of the device, a study was conducted to confirm the feasibility of using the device for kidney measurements. As an example, Figure 6a shows the bioimpedance data obtained on a porcine renal model, representing the complex bioimpedance as a function of frequency. The obtained values show clear agreement with the Cole model, which highlights both the validity of the measurements and the model itself. Figure 6b presents the bioimpedance data corresponding to a measurement performed on a human kidney, fifteen minutes after its excision during a complete nephrectomy, confirming the feasibility of using the device for measurements on human tissue.

### 3.3. Validation of the Accuracy and Repeatability of Bioimpedance Measurements in Renal Models

To study the repeatability of bioimpedance measurements on pig kidneys, at least three consecutive measurements were performed within a 5-min interval for the short-term repeatability assessment. For the long-term evolution assessment, at least 15 evaluations were performed within a 70-h ischemia period, ensuring a shorter interval between measurements in the first few hours. The measurements were performed on six pig kidneys on different dates.

The results of the repeatability analysis for localized measurements (see Figure 3) according to the procedure described in Section 2 highlighted significant variability (estimated via standard deviation) in successive measurements compared to evolving measurements, especially in the resistance at infinite frequency ($R_{\infty }$). This finding indicates low inherent repeatability in the measurements. It also suggests a considerable influence of electrode placement on the renal tissue in the obtained results. This circumstance limited the feasibility of conducting precise comparative studies of bioimpedance parameters at different time points.

To address the problem of repeatability in measurements, a new scheme was proposed based on longitudinal bioimpedance measurements over the entire renal organ, as opposed to localized measurements. As described in Section 3.1, the current injection electrodes are positioned at opposite ends of the organ, while the voltage measurement electrodes are located adjacent to the injection electrodes, also at the ends of the kidney. This configuration allows the global impedance of the renal organ to be the dominant factor in the measurement, significantly reducing the influence of the individual electrode placement on the renal tissue. By minimizing the dependence on the precise position of the electrodes, the repeatability of the measurements is improved, which is crucial for obtaining reliable data.

**Figure 7:**
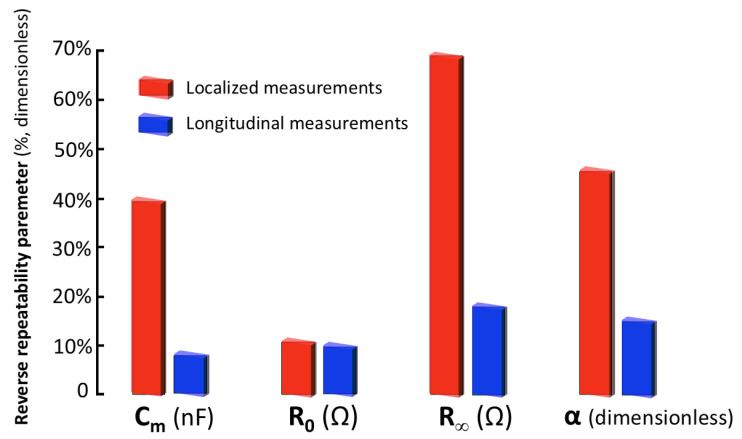
Comparative analysis of localized and longitudinal measurements by means of the reverse repeatability parameter (defined as in Section 2.5, the smaller the better) estimated for: resistance at zero frequency ($R_{0}$), resistance at infinite frequency ($R_{\inf}$), capacitive component associated with the membrane ($C_{M}$), and characteristic order of the frequency relaxation distribution (α) [47].

To facilitate the comparison, Figure 7 shows the reverse repeatability parameter (see Section 2.5) for the parameters of the Cole model, both for localized and longitudinal measurements. It is observed that the repeatability is greater in longitudinal measurements compared to localized ones. The results obtained confirm the suitability of the longitudinal electrode system for the acquisition of accurate and repeatable bioimpedance measurements in renal tissue. Short-term repeatability favors the significance of long-term parameter variation and facilitates the comparative analysis of parameters at different time points, allowing the identification of subtle changes in the bioelectrical properties of renal tissue.

### 3.4. Comparative Discussion of Accuracy and Repeatability with Bioimpedance Models

**Figure 8:**
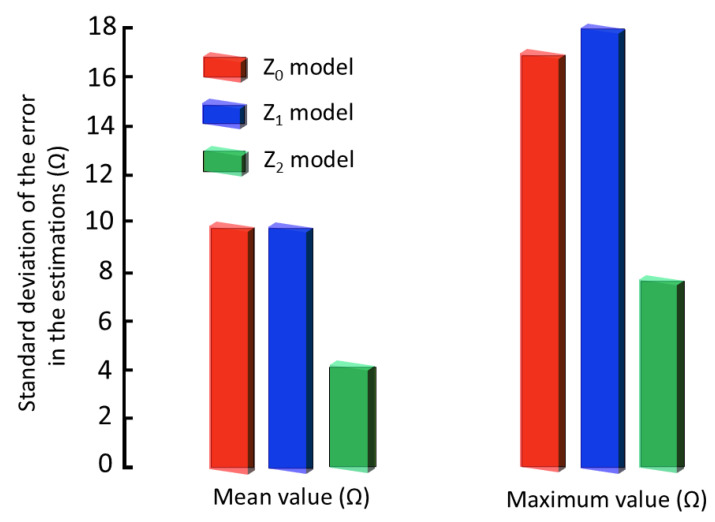
Comparative analysis of the fitting error of different bioimpedance models: Cole model ($Z_{0}$), model with parasitic capacitance ($Z_{1}$), and model with frequency-dependent membrane capacitance ($Z_{2}$).

The accuracy of the model parameters is intrinsically linked to the magnitude of the discrepancies observed between the experimental measurements and the model predictions. Therefore, a more precise approximation to the measured bioimpedance values translates into greater significance of the model parameters. Figure 8 presents the standard deviation, expressed in ohms, regarding both the mean values and maximum values as a quantitative measure of the error between the bioimpedance values predicted by the models and the experimentally obtained bioimpedance values. In the figure, it can be observed that the model corresponding to $Z_{1}$ does not exhibit a substantial improvement in the results compared to the Cole model. However, the new model $Z_{2}$ achieves a significant improvement in the model’s approximation to the bioimpedance values, evidenced by a 59% reduction in the estimation error compared to the generic Cole model ($p<10^{-6}$, paired *t*-test). By virtue of these substantial improvements in accuracy and error reduction, the $Z_{2}$ model is proposed as a new model for the characterization of bioimpedance measurements.

**Figure 9:**
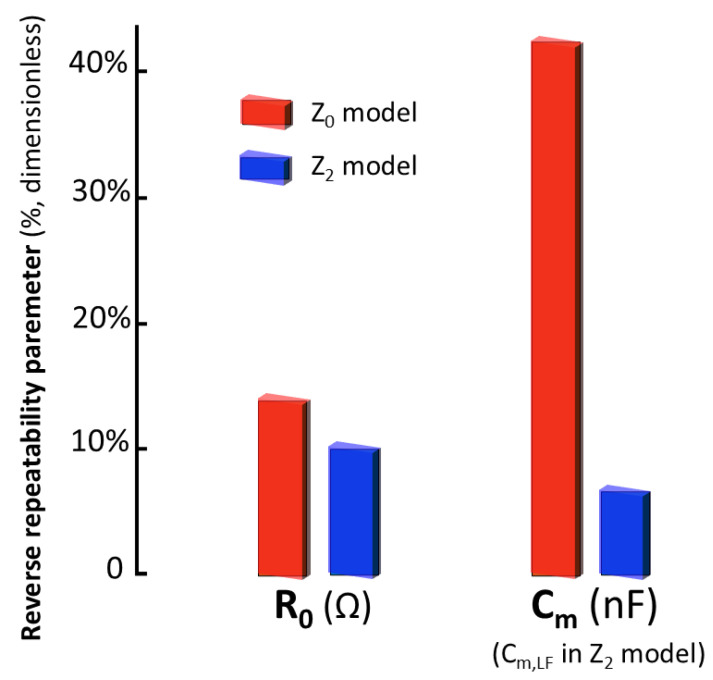
Comparative analysis of $Z_{0}$ and $Z_{2}$ models by means of the reverse repeatability parameter (defined as in Section 2.5, the smaller the better) estimated for: $R_{0}$ and $C_{m}$.

As previously established, to ensure the repeatability of the measurements, it is imperative that the standard deviation of successive measurements be significantly less than the standard deviation observed in the evolution study. This condition is fundamental to confer statistical significance to the variations detected in the parameters, in relation to the inherent measurement error. Figure 9 presents the reverse repeatability parameter, as defined previously in Section 2.5 for the most stable parameters of the $Z_{2}$ model in its dynamics ($R_{0}$ and $C_{m}$). According to these results, the $Z_{2}$ model exhibits a reverse repeatability in successive measurements of 7.5%, while the Cole $Z_{0}$ model presents a reverse repeatability of 42.1%. The significant improvement in repeatability achieved with the $Z_{2}$ model ($p<10^{-9}$, paired *t*-test) validates its superiority in characterizing the bioelectrical properties of renal tissue.

Figure 6 also highlights the errors committed in the $Z_{0}$ and $Z_{2}$ models that best fit the bioimpedance values. Indeed, the $Z_{2}$ model shows the greatest correspondence across the entire frequency range, while the $Z_{0}$ model suffers from a greater degree of discrepancies at low frequencies, but especially at high frequencies compensated with a greater importance of the $T_{D}$ value.

The comparative evaluation of the fitting error and the analysis of the temporal dynamics of bioimpedance parameters converge on a univocal conclusion: the proposed model exhibits a manifest suitability for the characterization of renal tissue through bioimpedance measurement. The observed concordance between the results validates the effectiveness of the proposed model as a tool for the precise estimation of the bioelectrical properties of the kidney. The robustness of the model is manifested in its ability to minimize discrepancies between theoretical predictions and experimental measurements, as well as in its sensitivity to detect subtle variations in the bioelectrical properties of the tissue over time.

### 3.5. Analysis of the Temporal Evolution of Bioimpedance Parameters

**Figure 10:**
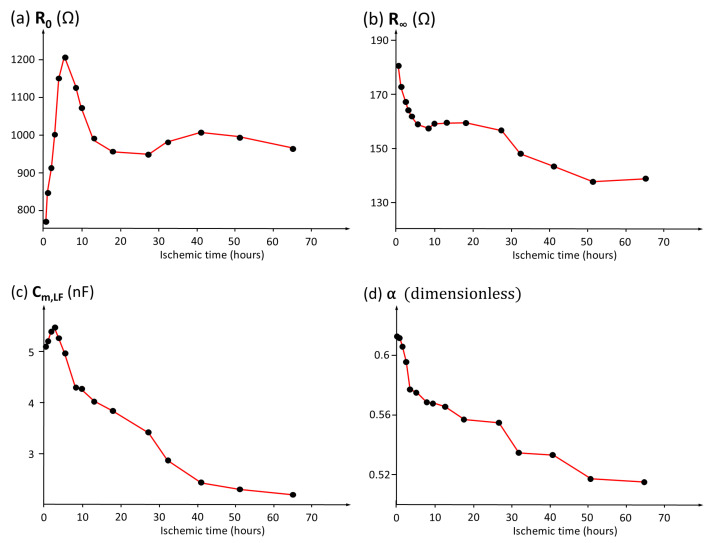
Temporal variation in bioimpedance parameters in a porcine renal model preserved in cold organ preservation solution: (**a**) $R_{0}$. (**b**) $R_{\infty }$. (**c**) $C_{m}$ (low frequency). (**d**) α.

Results of interest have also been obtained from the analysis of the evolution of bioimpedance parameters in pig kidneys preserved in cold organ preservation fluid. Figure 10a–d show the details of the results obtained in a porcine renal model during a period of ischemia. According to the results presented, the most stable parameter in its dynamics is the membrane capacitance at low frequency. In this parameter, rapid growth is observed in the first two hours of preservation, subsequently initiating a progressive decrease to a stationary value. Another parameter with a well-defined curve is the α parameter, related to the dispersion of membrane capacitance values around the mean value, since cells have different sizes and belong to different tissues. The curve indicates that there is a decrease in the dispersion of capacitance values and in cell heterogeneity as ischemia progresses. The decrease in $R_{\infty }$, which would be expected to remain stable if the kidney were isolated, indicates a progressive accumulation of fluids, since the kidney is immersed in organ preservation fluid in the time between measurements.

Both $R_{0}$ and $C_{m}$ show significant increases during the first hours of ischemia, followed by progressive decreases until reaching stationary values. These results agree with the hypotheses proposed by other researchers [53], who suggest that organ extraction induces an ischemic cellular inflammatory response, resulting from the inhibition of energy metabolism. This inflammatory response leads to a reduction in the extracellular space, which in turn decreases the width of the electrical path for low-frequency currents, resulting in an increase in $R_{0}$. Similarly, cellular edema causes an increase in membrane capacitance ($C_{m}$) due to the increase in the cell membrane surface area. Finally, as cell death progresses, the rupture of cell membranes occurs, resulting in an increase in extracellular space and consequent decreases in $R_{0}$ and $C_{m}$. The agreement of the experimental results with the previously established hypotheses validates the ability of the proposed bioimpedance model to monitor the pathophysiological changes associated with renal ischemia.

## 4. Discussion

A prototype of an intelligent bioimpedance spectroscopy device has been developed and validated, designed for the objective evaluation of renal graft viability in the context of transplantation. The results obtained with this preliminary prototype demonstrate the accuracy and feasibility of the proposed technology, as well as its potential for clinical applications. The accuracy of the prototype, validated by measuring a circuit pattern with components of known values, yielded an error of less than 1%, a result comparable to those obtained in similar systems reported in the scientific literature [54]. The use of a circuit pattern with resistors and capacitors of predefined parameters is essential for the objective evaluation of the accuracy of the estimates, given the impossibility of knowing a priori the bioimpedance values of renal tissue.

The results obtained with the prototype confirm the feasibility of the proposed technology for the acquisition of bioimpedance measurements in the context of renal transplantation. The proposed device offers the ability to provide quantitative measurements complementary to biopsy, with the aim of optimizing decision-making in the renal transplantation process. The integration of this technology into the clinical workflow could increase the efficiency of the process, improving the chances of patients receiving a viable renal graft, reducing the rate of discarded organs, and decreasing waiting lists. These improvements would have a positive impact both socially and economically for the Public Health System, by optimizing resource allocation and improving clinical outcomes.

Despite the demonstrated clinical utility of bioimpedance analysis for the objective evaluation of the condition and viability of organs intended for transplantation, its routine clinical application is hindered by unresolved research challenges. Consequently, bioimpedance research has been predominantly limited to studies regarding animal models or human organs discarded after surgical excision procedures. One of the main causes of this limitation lies in the lack of repeatability in the estimation of bioimpedance parameters. In the present work, this problem is addressed by proposing a new bioimpedance model that significantly improves the fit to experimental data, reducing estimation error and increasing repeatability in the estimation of model parameters. This improvement represents a significant advancement in relation to the existing models in the literature. The key innovation of the proposed model resides in the introduction of a frequency-dependent membrane capacitance, a novel concept in the context of bioimpedance measurements. This frequency dependence opens new avenues of research to understand the underlying mechanisms that modulate the electrical properties of cell membranes. The model validation has been performed through experimental measurements in porcine renal models, demonstrating its superiority in terms of repeatability (short-term measurements) and dynamic significance (long-term measurements) compared to other existing models.

The results shown in the work support the clinical utility of bioimpedance analysis, as evidenced in the evaluation of temporal evolution presented in Figure 10, which captures the changes observed during the ischemia process. In this figure, a rapid increase in the resistance associated with the extracellular compartment is observed during the initial phase of the ischemia process. These findings are consistent with the results reported by other researchers in studies conducted on hepatic tissue samples [37,55], where a similar phenomenon related to cellular edema induced by osmotic imbalance has been documented.

In [37], an in-depth study of the evolution of bioimpedance parameters during ischemia in a rabbit liver at 37 °C was carried out. This study highlighted a rapid increase in $R_{0}$ at the onset of ischemia, reaching a peak at approximately 30 min and decreasing thereafter. The authors associated this fact with a change in metabolic activities. By separating the organ from the body circulation, the lack of oxygen and nutrients caused the rupture of the ionic pumps in the cell membrane. The accumulation of intracellular fluid, as a consequence of the alteration in osmotic gradients across the cell membrane, results in an increase in cell volume and the consequent compression of the extracellular space. The intracellular osmotic pressure increased, while the extracellular ionic concentration decreased, causing an increase in cell volume and the consequent compression of the extracellular space. This compression manifests as an increase in extracellular resistance, reflecting the decrease in the cross-sectional area available for the flow of electrical current through the extracellular compartment. After 30 min, the gradual destruction of the cell membrane reduced the electrical impedance. This change in bioimpedance values was associated with the loss of cell membrane integrity and, consequently, with the point of no return for organ viability. After 5 h, the impedance decreased significantly, indicating that at that point the cell membranes had been severely damaged and tissue activity had been considerably reduced. This hypothesis was corroborated by the cellular morphological changes observed through microscopy of tissue sections: the cells first swelled, then the cytoplasm became slightly stained, and subsequently, the cell membrane gradually dissolved, resulting in an irregular cell shape and an expansion of the extracellular space. Finally, the nucleus collapsed and disappeared, until all cellular activity was completely lost. After 5 h, the number of nuclei were markedly reduced, suggesting severe cell damage. During devitalization, the cells acquired an irregular shape, and the intercellular area increased due to the disruption of the cell membrane and cytoskeleton.

These morphological results were also reported by [56], who found that, once human liver was isolated at 37 °C, the tissue viability began to deteriorate. As the ex vivo time increased, the liver injury gradually worsened, and the cell morphology changed, including vacuolation, cytolysis, and cell swelling. When liver tissue was isolated, not only did the nucleus expand but the cell swelled. One hour after isolation, nuclear pyknosis occurred, and the nuclear area gradually decreased. The cell nuclei shrank as the isolation time prolonged. At 8 h, the expansion of the intercellular space led to the rupture of the cell membrane, leakage of intracellular fluid, and gradual cell ablation.

Similarly, the results of [57] indicate a substantial increase in the impedance modulus throughout the warm ischemic period in rat kidneys, attributed to the onset of hypoxic edema as a result of cellular swelling, leading to a reduction in the extracellular space, an increase in extracellular resistance, and intercellular uncoupling. After unclamping of the renal artery at 50 min, the impedance modulus was observed to return to its baseline value, which in this experimental context can be attributed to reperfusion without substantial structural damage to the tissue. Failure of reperfusion is associated with a decrease in the impedance modulus at low frequencies as a consequence of membrane rupture and cell lysis due to sustained ischemia.

Another related study is [58], which investigated changes in human intestinal bioimpedance noninvasively during ischemia. After resection, the impedance increased over the next two hours and continued to decrease until the end of the experiment. The time interval at which it reached its maximum was consistent with the viability/non-viability limits reported in the histological analysis.

In [59] the authors showed the evolution of pig liver impedance at 36 °C, with an almost linear increase in impedance at low frequency after excision. Furthermore, they were able to verify that this rate of increase decreased if the organ was cooled, which is consistent with the results shown in this work, with the inflection instant of behavior delayed in time.

These results have been widely corroborated in the literature. According to [60], rat liver bioimpedance changed significantly during low-frequency ischemia compared to high-frequency ischemia, returning to its baseline level after reperfusion. In [61], apoptosis was identified in the gastric mucosa of rats starting after 90 min of ischemia, with resistance to low frequencies being related to damage, which allowed predicting the occurrence of reversible and irreversible tissue damage. The same results were observed in [62] on bioimpedance measurements during ischemia in rat liver, with a tendency for a gradual increase after ischemia and a return to the baseline levels after reperfusion. The objective of [63] was to determine whether ischemic and reperfusion damage in cardiac surgery could be detected by bioimpedance. Low-frequency bioimpedance increased during ischemia, and then the values returned almost to the baseline measurements. The in vivo bioimpedance analysis in a model of global cerebral ischemia in anesthetized rats also suggests that impedance increases steadily during occlusion.

The referenced authors propose the identification of a point of no return, from which organ recovery is considered unfeasible. This critical point is defined as the instant at which the exponential increase phase of extracellular resistance concludes. The observation of this phenomenon, characterized by an abrupt and sustained elevation in extracellular resistance, suggests the utility of the bioimpedance technique as a tool for evaluating the state of ischemia and detecting the progression towards cell death in the renal organ. The ability of bioimpedance to monitor changes in extracellular resistance, a sensitive indicator of cellular integrity and tissue viability, allows for the early identification of pathophysiological alterations associated with ischemia. This early identification is crucial for timely clinical decision-making and the implementation of more effective organ preservation strategies.

Future work is planned to overcome some of the limitations/lessons learned from the translation of the bioimpedance device to the surgical setting, such as the difficulty in using insulating elements in direct contact with the organ. In this case, the use of a plastic surface was not feasible, and it was decided to use sterile gauze on a stainless steel surface, so the immunity of the measurements to the conductive surface must be ensured. On the other hand, the procedure for inserting sterilized electrodes into the sterile sheath covering the probe and cables should also be reviewed as insulating elements can be introduced to prevent internal contact with the electrodes. Furthermore, plans are underway to expand the number of measurements to confirm the results, integrate the system into daily clinical practice, and use bioimpedance measurements alongside other clinical variables to complement a more objective assessment of graft status and the analysis of potential relationships between bioimpedance measurements and clinical data for the investigation of new biomarkers for kidney graft viability. As in the present work human kidney measurements were limited to excised organs; future studies will validate the device in live transplant settings.

## 5. Conclusions

The developed bioimpedance spectroscopy device represents a promising tool for the objective evaluation of renal graft viability. Its ability to provide accurate and repeatable quantitative measurements, along with the improvement in bioimpedance modeling, could optimize clinical decision-making in renal transplantation. The integration of this technology into the clinical workflow has the potential to reduce the rate of discarded organs, decrease waiting lists, and improve clinical outcomes for patients.

The adaptation of the device for use in the operating room required overcoming significant obstacles, mainly sterilization and the design of a specialized user interface. The implementation of a strict sterilization protocol for the electrodes, which includes the selection of materials compatible with the human body and the validation of autoclave sterilization methods, was essential to ensure patient safety and reduce the risk of nosocomial infections.

The new proposed bioimpedance model, with its frequency-dependent membrane capacitance, represents a significant advancement in the characterization of renal tissue. Its superiority in terms of repeatability, validated in studies with porcine renal models, opens new avenues of research to understand the underlying mechanisms that modulate the electrical properties of biological media.

The ability of the device to monitor the pathophysiological changes associated with renal ischemia, evidenced in the analysis of the temporal evolution of bioimpedance parameters, suggests its clinical utility in the objective evaluation of the state and viability of organs intended for transplantation. The identification of a point of no return in organ recovery, based on changes in extracellular resistance, could improve organ preservation strategies and timely clinical decision-making.

## Figures and Tables

**Figure A1:**
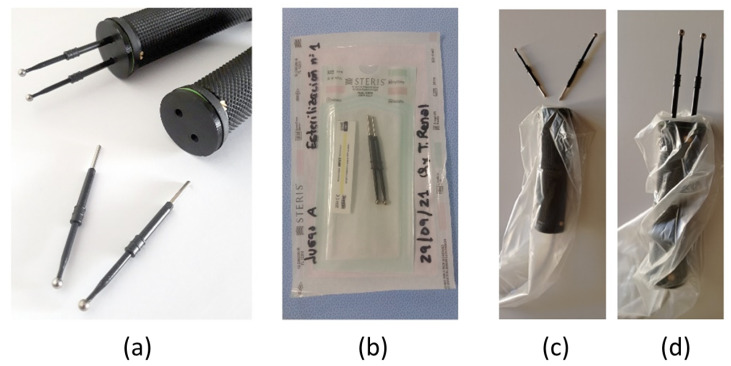
(**a**) Details of the electrodes used in the operating room. (**b**) Packaging for sterilization control. (**c**,**d**) Sterile cover and electrode insertion procedure.

## Data Availability

The data presented in this study are available on request from the corresponding author.

## Appendix A. Design of the Measurement Protocol in the Operating Room

In consensus with the clinical team, a measurement protocol was established in the operating room, which largely determined the user interface of the device. This protocol will include two measurements once the device is incorporated into routine transplant procedures in the operating room (although some tests of use of the device following the protocol have already been carried out): (1) An initial measurement of the organ after extraction. (2) A final measurement of the organ just before implantation. In addition to bioimpedance measurements, measurements of organ length, central perimeter, and weight must be taken. The control device should be placed on an auxiliary table near the work area. The measurement device, cable, and probe should be covered with a sterile sheath similar to that used for other devices in the operating room (see Figure A1c). Before use, the electrodes must have been sterilized in an autoclave following the manufacturer’s recommendations. The sterilized electrodes must be stored appropriately to ensure the preservation of sterilization (see Figure A1b). When they are to be used, they will be connected to the probes covered with the sterile sheath, ensuring sterilization (see Figure A1d). Spare units of the electrodes will be available in case of any eventuality. The measurement process is carried out by the clinical team on a sterile auxiliary work table, removing excess moisture with a sterile gauze and placing the organ on a sterilized non-conductive surface.

Given the complexity of all these processes and the need for verification by the Central Sterilization Department so that bioimpedance measurements can be safely incorporated into transplant surgical procedures, it has also been necessary to establish a Standard Operating Procedure for the sterilization of the electrodes. This protocol has been defined in a technical document approved by the Central Sterilization Department, which includes, among other things, the following specifications:

- Elements to be sterilized: The electrodes selected for use in the operating room are the FIAB F4068 commercial electrosurgery electrodes described in Section 3.1. Four new sterilized electrodes will be needed for each measurement (see Figure A1a). In successive measurements, carried out in a short period of time, the same set of electrodes can be used, but they will be renewed in the next measurement.
- Sterilization method: Following the recommendations of the manufacturer’s technical data sheet and in accordance with current regulations, the maximum number of sterilization cycles (20 cycles) and the exposure times, pressure, and temperatures of the autoclave sterilization process are established following the manufacturer’s instructions (for 134 °C, 2.05 bar, and 12 min; for 121°C, 1.05 bar, and 20 min).
- Packaging procedure: Each pair of electrodes will be packaged in self-adhesive bags for steam or ethylene oxide sterilization individually, and both bags will in turn be placed together in a second mixed bag. Each unit package of the electrode will carry an internal steam chemical control (see Figure A1b).
- Sterilization cycle control: In the Central Sterilization Department, a record will be kept with the control of the sterilization cycles carried out. Each package will be identified with the corresponding sterilization cycle number. After use and before sending them back to the Central Department, the operating room staff must identify on the package the next sterilization cycle that would correspond to it. If, in any case, the four electrodes of each package are sent to the sterilization department without the identification of the package and the number of previous sterilization cycles carried out, for safety reasons, their sterilization will not proceed, considering the useful life of said electrodes to be over.
